# Supplementary material for: Genome mining shows that retroviruses are pervasively invading vertebrate genomes
Source: Nat Commun. 2023 Aug 17;14:4968. doi: 10.1038/s41467-023-40732-w (PMC10435555; doi:10.1038/s41467-023-40732-w)
Supplement: Supplementary file 1 — SUPPLEMENTARY INFO [file 41467_2023_40732_MOESM1_ESM.pdf]

Genome mining shows that retroviruses are pervasively invading vertebrate genomes

Jianhua Wang[1], Guan-Zhu Han[1*]

[1]College of Life Sciences, Nanjing Normal University, Nanjing, China

[*] To whom correspondence should be addressed. E-mail: guanzhu@njnu.edu.cn.

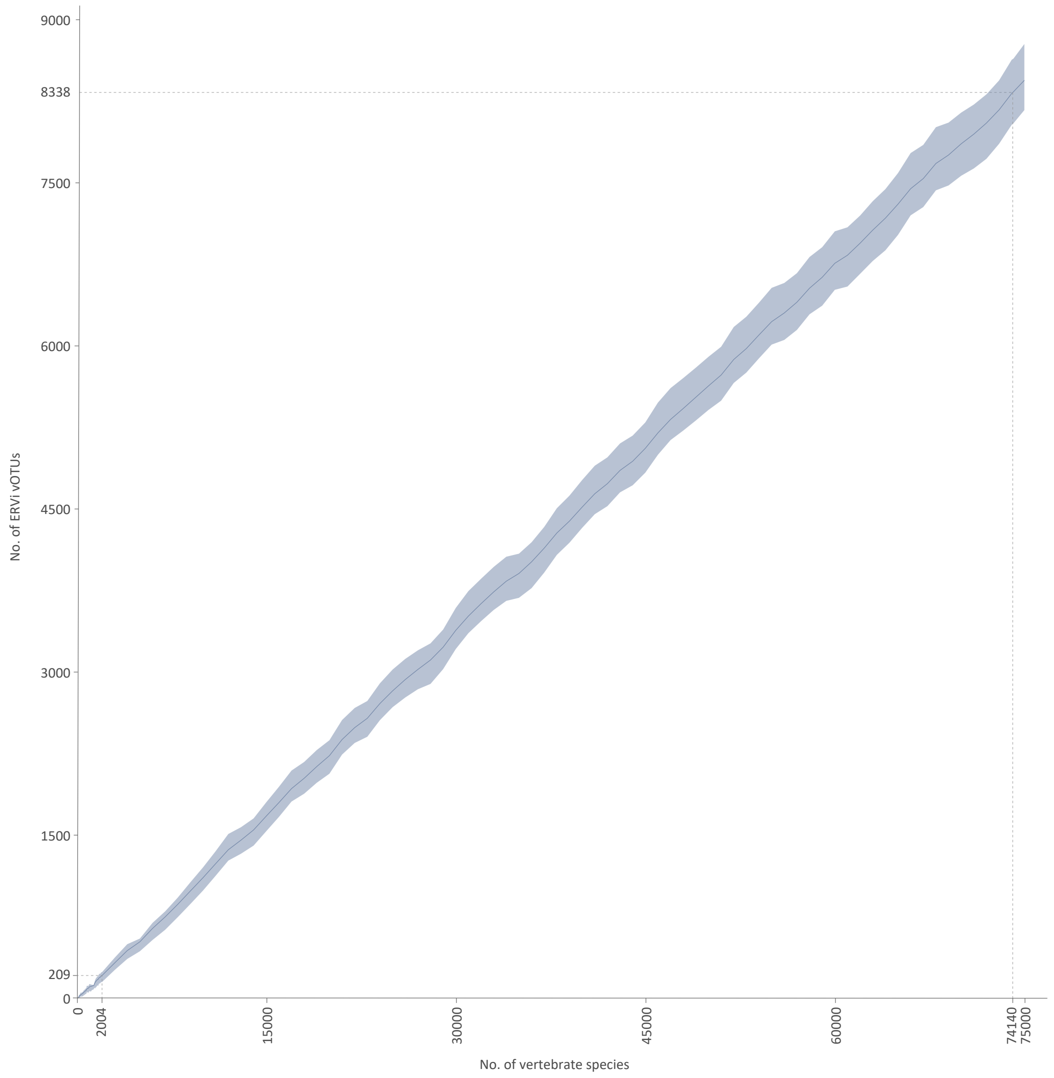

Supplementary Fig. 1 The simulation of ERVi vOTU numbers for different numbers of vertebrate species. The center line represents the observed value of the number of ERVi vOTUs for the number of corresponding vertebrate species. The blue shadow represents 95% confidence intervals estimated using the BCa method with 10,000 bootstrap replicates. Abbreviations: viral operational taxonomic units (vOTUs).

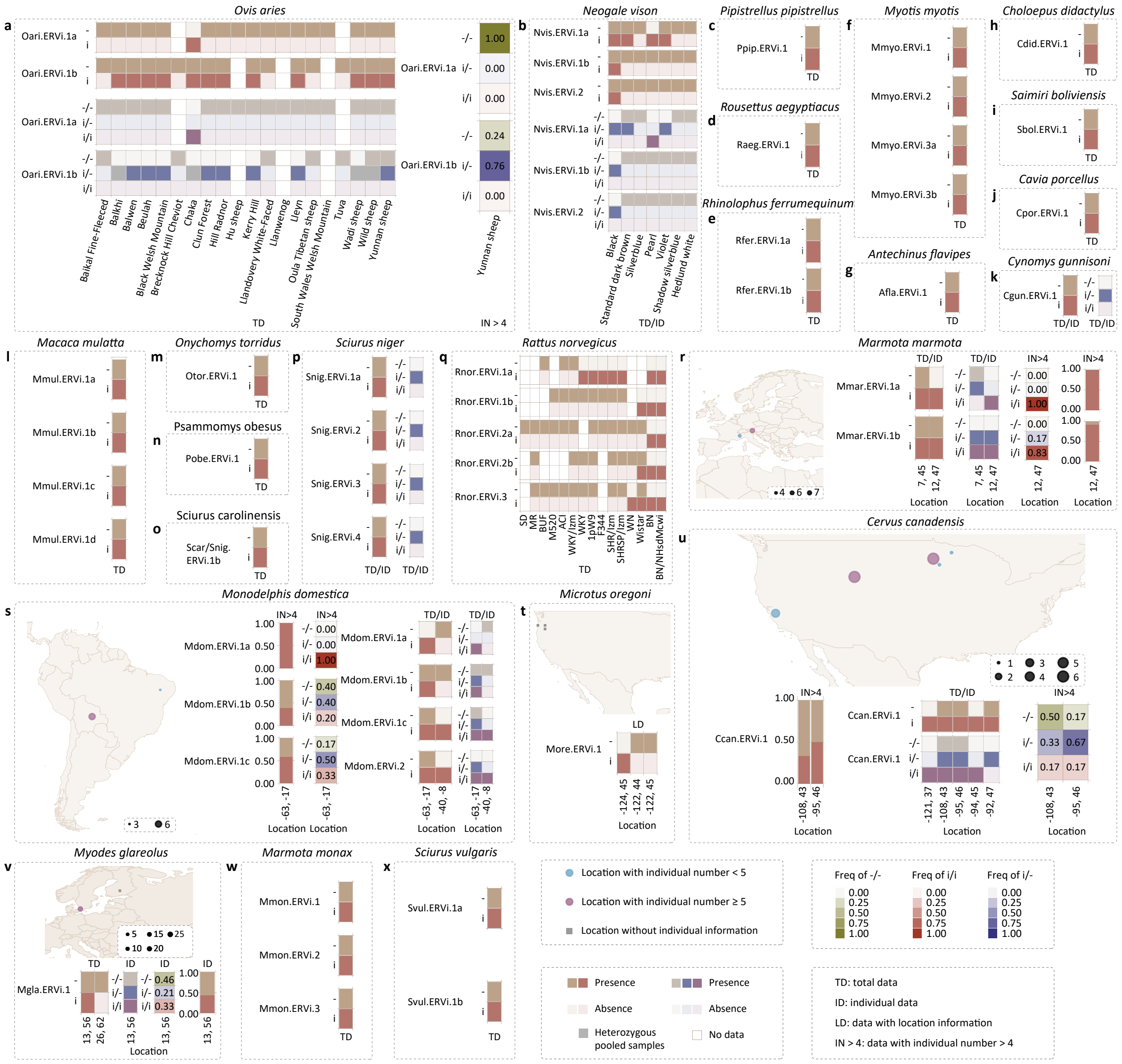

Supplementary Fig. 2 The geographic distribution and prevalence of ERVi in mammals. **a**, *Ovis aries*. **b**, *Neogale vison*. **c**, *Pipistrellus pipistrellus*. **d**, *Rousettus aegyptiacus*. **e**, *Rhinolophus ferrumequinum*. **f**, *Myotis myotis*. **g**, *Antechinus flavipes*. **h**, *Choloepus didactylus*. **i**, *Saimiri boliviensis*. **j**, *Cavia porcellus*. **k**, *Cynomys gunnisoni*. **l**, *Macaca mulatta*. **m**, *Onychomys torridus*. **n**, *Psammomys obesus*. **o**, *Sciurus carolinensis*. **p**, *Sciurus niger*. **q**, *Rattus norvegicus*. **r**, *Marmota marmota*. **s**, *Monodelphis domestica*. **t**, *Microtus oregoni*. **u**, *Cervus canadensis*. **v**, *Myodes glareolus*. **w**, *Marmota monax*. **x**, *Sciurus vulgaris*. For each species, diverse datasets were used to identify the presence (i) and absence (-) of ERVi insertion in a locus: TD (total data) include all the evidentiary genome sequencing data of individuals and samples without detailed individual information; ID (individual data) include evidentiary genome sequencing data for individuals; LD represents evidentiary genome sequencing data with location information. For each species with

ID data, genotypes i/i, i/-, and -/- were shown. For (**a**) pattern, TD data was used to show the presence and absence of i/i, i/-, and -/-, and samples without detailed individual information that can be inferred to be heterozygous (i/-) were labeled in gray. For each species or area grid cell (~ 12,321 km^2), when population genomics data for more than 4 individuals (IN > 4) are available, the frequency of genotype (i/i, i/- and -/-) were shown. Circle size indicates sample size in the corresponding location. Locations with individual number < and ≥ 5 are labeled in blue and purple, respectively. Locations without detail individual information are labeled with gray rectangles.

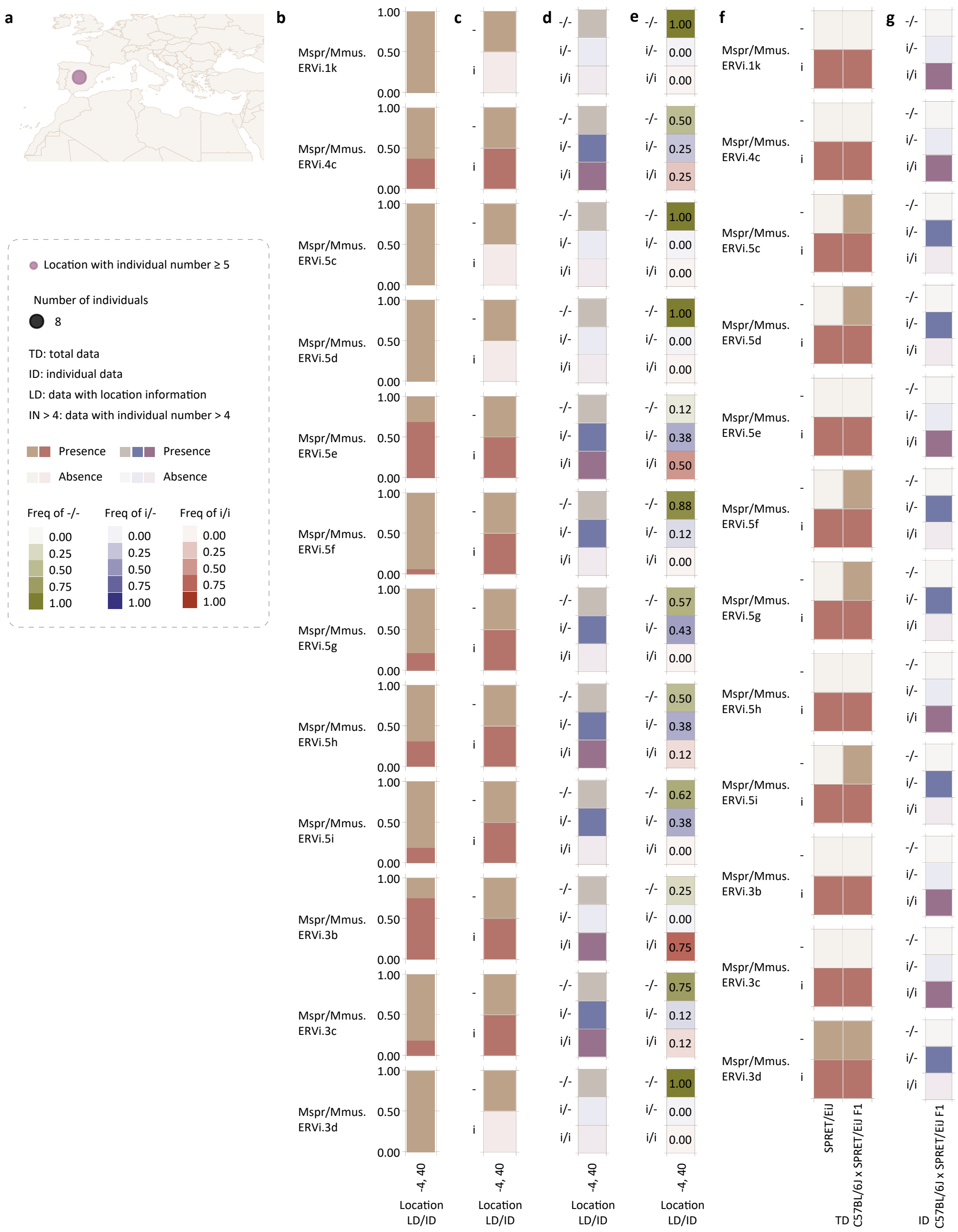

Supplementary Fig. 3 The geographic distribution and prevalence of Mspr/Mmus.ERVi. a, The geographic distribution of sampling points for *Mus spretus*. Circle size indicates sample size in the corresponding location. Locations with individual number ≥ 5 are labeled in purple. **b**, The frequency of Mspr/Mmus.ERVi insertion (i) and empty locus (-). **c**, The presence (i) and absence (-) of Mspr/Mmus.ERVi. **d**, The presence and absence of different genotype (i/i, i/-, and -/-) for Mspr/Mmus.ERVi. **e**, The genotype (i/i, i/-, and -/-) frequency of Mspr/Mmus.ERVi. TD (total data) include all the evidentiary genome sequencing data of individuals and samples without detailed individual information; ID (individual data) include evidentiary genome sequencing data for individuals; LD represents evidentiary genome sequencing data with location information. ID

represents evidentiary individual sequencing data of wild (**b**-**e**) or laboratory strains (**g**) for *Mus spretus*.

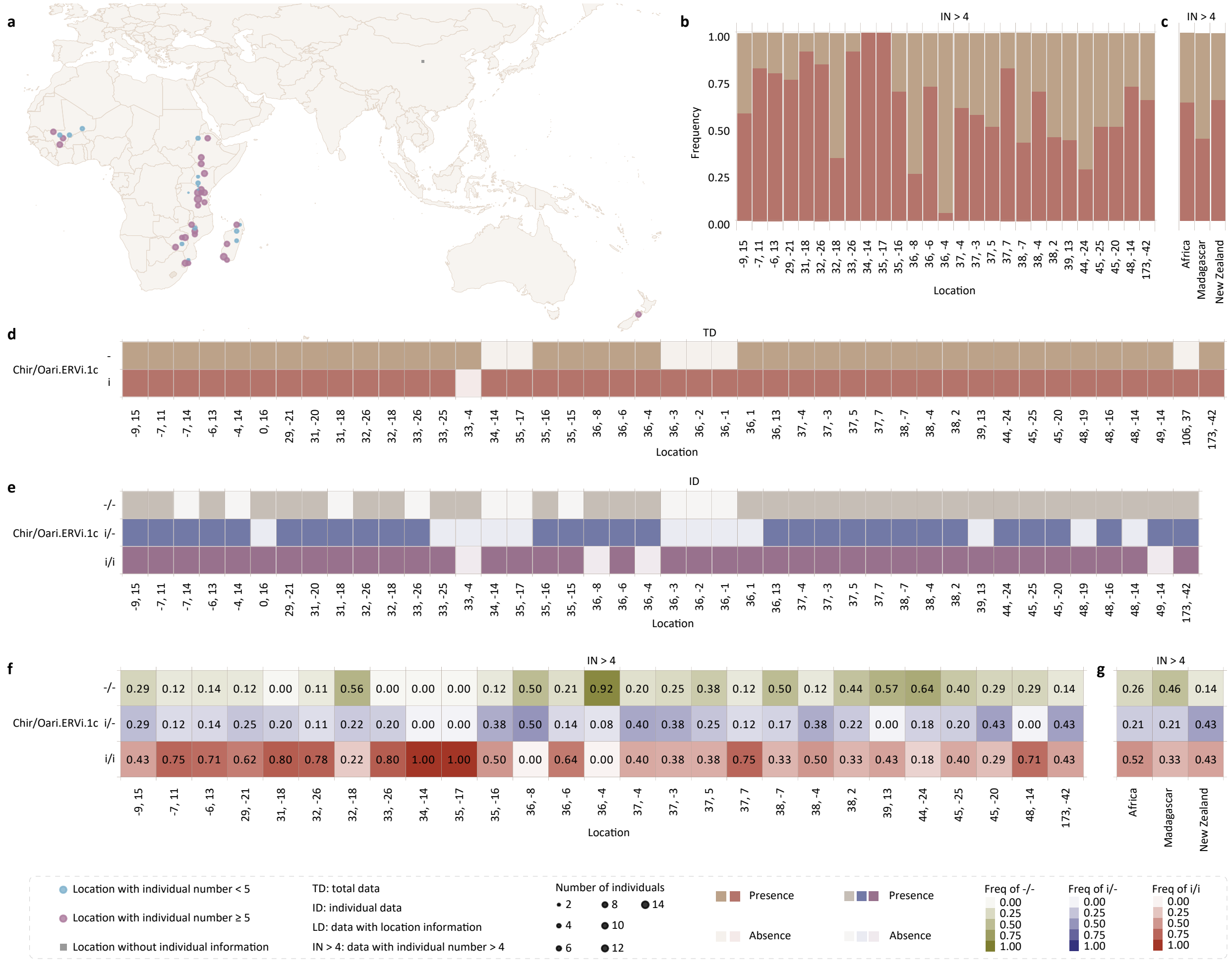

Supplementary Fig. 4 The geographic distribution and prevalence of Chir/Oari.ERVi. a, The spatial distribution of sampling points for *Capra hircus*. Circle size indicates sample size in the corresponding location. Locations with individual number < and ≥ 5 are labeled in blue and purple, respectively. Locations without detail individual information are labeled with gray rectangles. **b**, The frequency of Chir/Oari.ERVi insertion (i) and empty locus (-) in different area grid cells (~ 12,321 km^2). **c**, The frequency of Chir/Oari.ERVi insertion (i) and empty locus (-) in different geographic regions. **d**, The presence (i) and absence (-) of Chir/Oari.ERVi in different area grid cells (~ 12,321 km^2). **e**, The presence and absence of different genotype (i/i, i/-, and -/-) for Chir/Oari.ERVi in different area grid cells (~ 12,321 km^2). **f**, The genotype (i/i, i/-, and -/-) frequency of Chir/Oari.ERVi in different area grid cells (~ 12,321 km^2). **g**, The genotype (i/i, i/-, and -/-) frequency of Chir/Oari.ERVi in different geographic regions. TD (total data) include all the evidentiary genome sequencing data of individuals and samples without detailed individual information; ID (individual data) include evidentiary genome sequencing data for individuals; IN > 4 represent an area grid cell or geographic region with the population genomics data for more than 4 individuals are available.

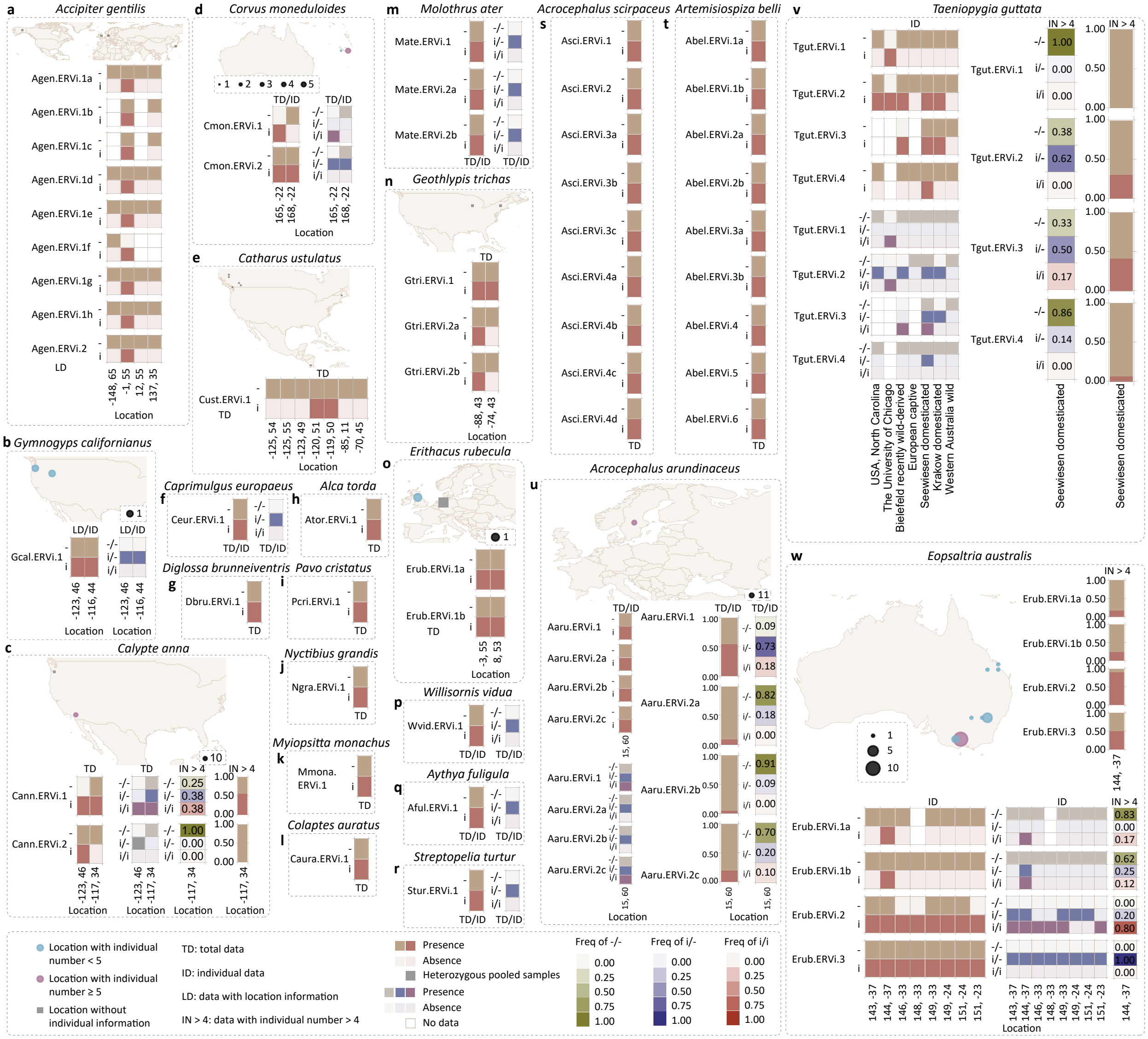

Supplementary Fig. 5 The geographic distribution and prevalence of ERVi in birds. a, *Accipiter gentilis*. **b,** *Gymnogyps californianus*. **c**, *Calypte anna*. **d**, *Corvus moneduloides*. **e**, *Catharus ustulatus*. **f**, *Caprimulgus europaeus*. **g**, *Diglossa brunneiventris*. **h**, *Alca torda*. **i**, *Pavo cristatus*. **j**, *Nyctibius grandis*. **k**, *Myiopsitta monachus*. **l**, *Colaptes auratus*. **m**, *Molothrus ater*. **n**, *Geothlypis trichas*. **o**, *Erithacus rubecula*. **p**, *Willisornis vidua*. **q**, *Aythya fuligula*. **r**, *Streptopelia turtur*. **s**, *Acrocephalus scirpaceus*. **t**, *Artemisiospiza belli*. **u**, *Acrocephalus arundinaceus*. **v**, T*aeniopygia guttata*. **w**, *Eopsaltria australis*. For each species, diverse datasets were used to identify the presence (i) and absence (-) of ERVi insertion in a locus: TD (total data) include all the evidentiary genome sequencing data of individuals, pooled individuals with or without sample size and samples without detailed individual information; ID (individual data) include evidentiary genome sequencing data for individuals and pooled individuals with sample size that can be inferred to be homozygous (i/i or -/-); LD represents evidentiary genome sequencing data with

location information. For each species with ID data, genotypes i/i, i/-, and -/- were shown. For (**c**) pattern, TD data was used to show the presence and absence of i/i, i/-, and -/-, and samples without detailed individual information that can be inferred to be heterozygous (i/-) were labeled in gray. For each species or area grid cell (~ 12,321 km^2), when population genomics data for more than 4 individuals (IN > 4) are available, the frequency of genotype (i/i, i/- and -/-) were shown. Circle size indicates sample size in the corresponding location. Locations with individual number < and ≥ 5 are labeled in blue and purple, respectively. Locations without detail individual information are labeled with gray rectangles.

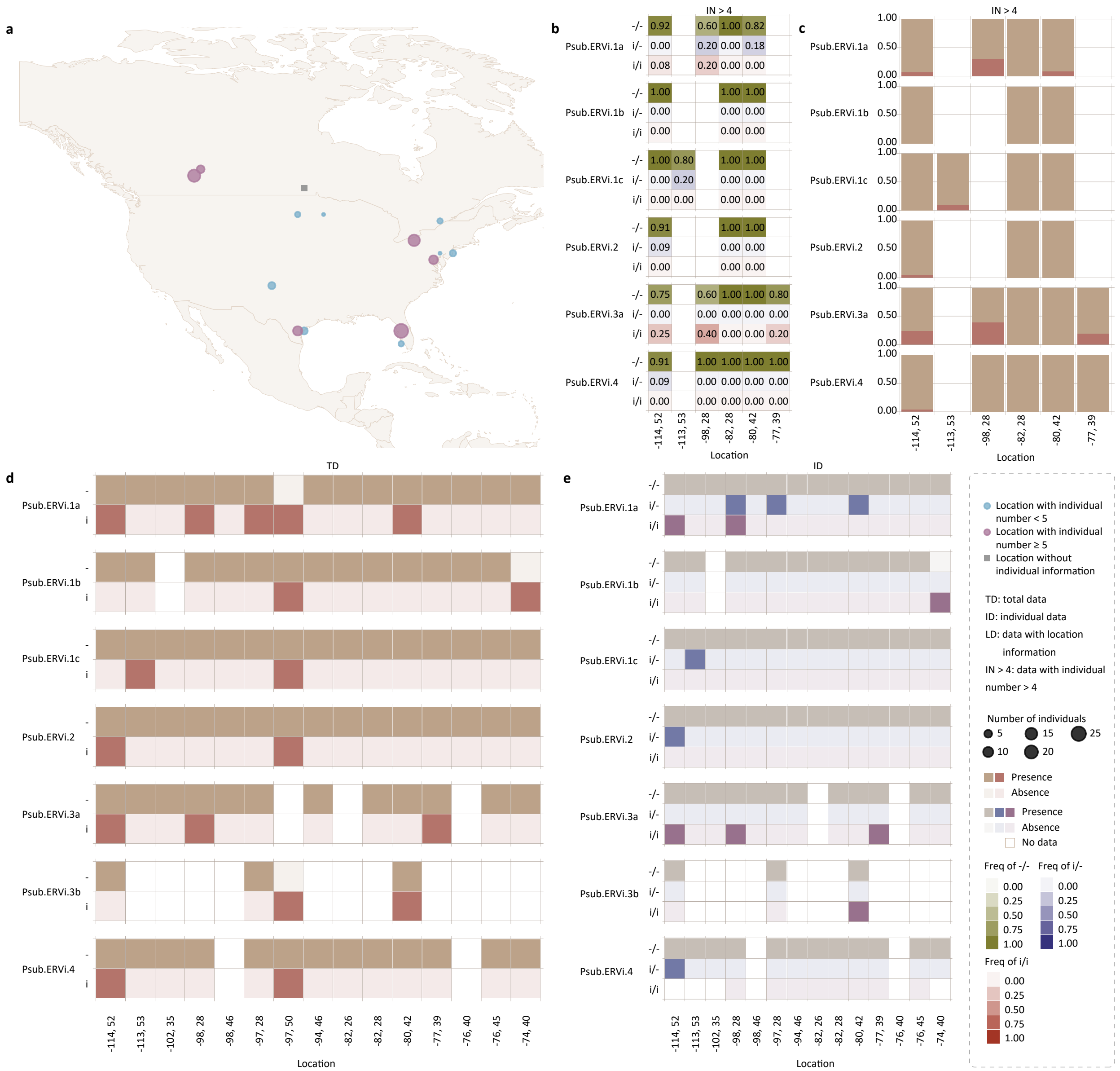

Supplementary Fig. 6 The geographic distribution and prevalence of Psub.ERVi. a, The geographic distribution of sampling points for *Progne subis*. Circle size indicates sample size in the corresponding location. Locations with individual number < and ≥ 5 are labeled in blue and purple, respectively. Locations without detail individual information are labeled with gray rectangles. **b**, The genotype (i/i, i/-, and -/-) frequency of Psub.ERVi in different area grid cells (~ 12,321 km^2). **c**, The frequency of Psub.ERVi insertion (i) and empty locus (-) in different area grid cells (~ 12,321 km^2). **d**, The presence (i) and absence (-) of Psub.ERVi in different area grid cells (~ 12,321 km^2). **e**, The presence and absence of different genotype (i/i, i/-, and -/-) for Psub.ERVi in different area grid cells (~ 12,321 km^2). TD (total data) include all the evidentiary genome sequencing data of individuals and samples without detailed individual information; ID (individual

data) include evidentiary genome sequencing data for individuals; IN > 4 represent an area grid cell with the population genomics data for more than 4 individuals are available.

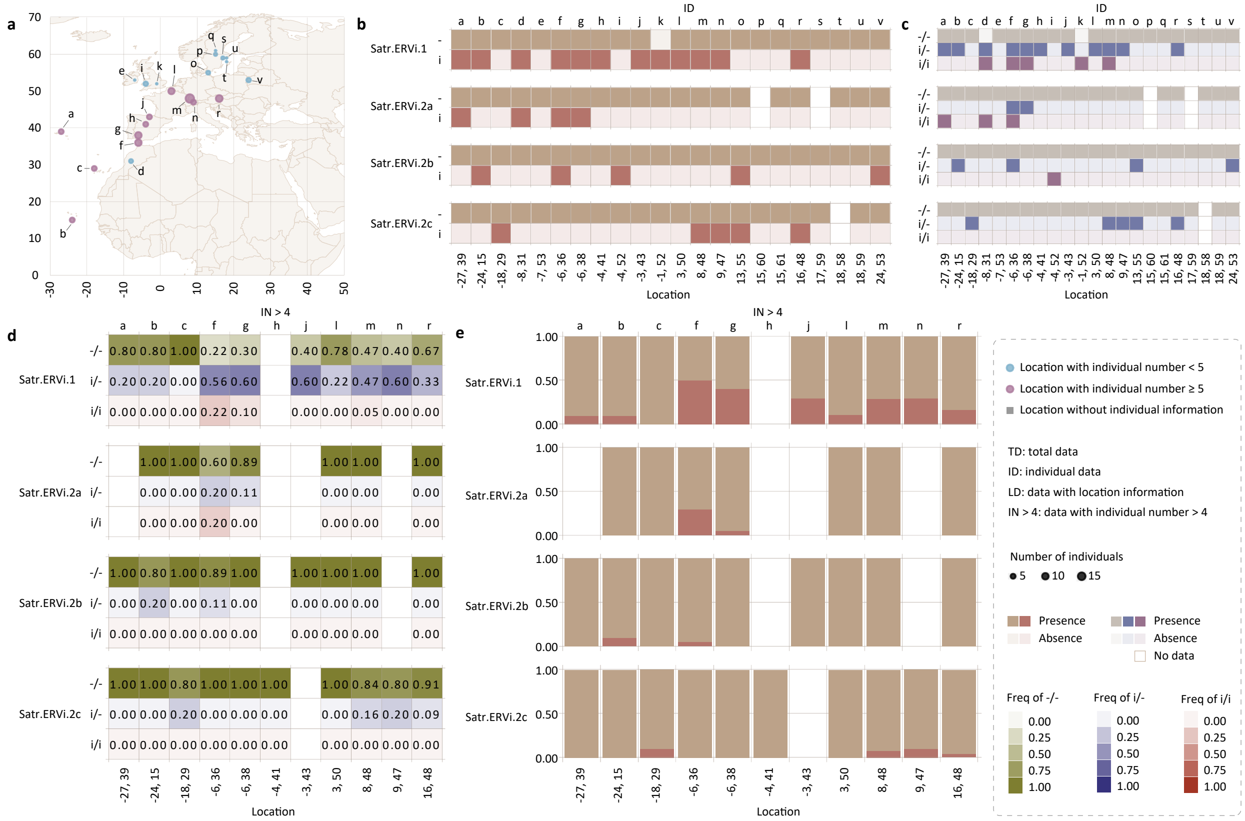

Supplementary Fig. 7 The geographic distribution and prevalence of Satr.ERVi. a, The geographic distribution of sampling points for *Sylvia atricapilla*. Circle size indicates sample size in the corresponding location. Locations with individual number < and ≥ 5 are labeled in blue and purple, respectively. **b**, The presence (i) and absence (-) of Satr.ERVi in different area grid cells (~ 12,321 km^2). **c**, The presence and absence of different genotype (i/i, i/-, and -/-) for Satr.ERVi in different area grid cells (~ 12,321 km^2). **d**, The genotype (i/i, i/-, and -/-) frequency of Satr.ERVi in different area grid cells (~ 12,321 km^2). **e**, The frequency of Satr.ERVi insertion (i) and empty locus (-) in different area grid cells (~ 12,321 km^2). ID (individual data) include evidentiary genome sequencing data for individuals; IN > 4 represent an area grid cell with the population genomics data for more than 4 individuals are available.

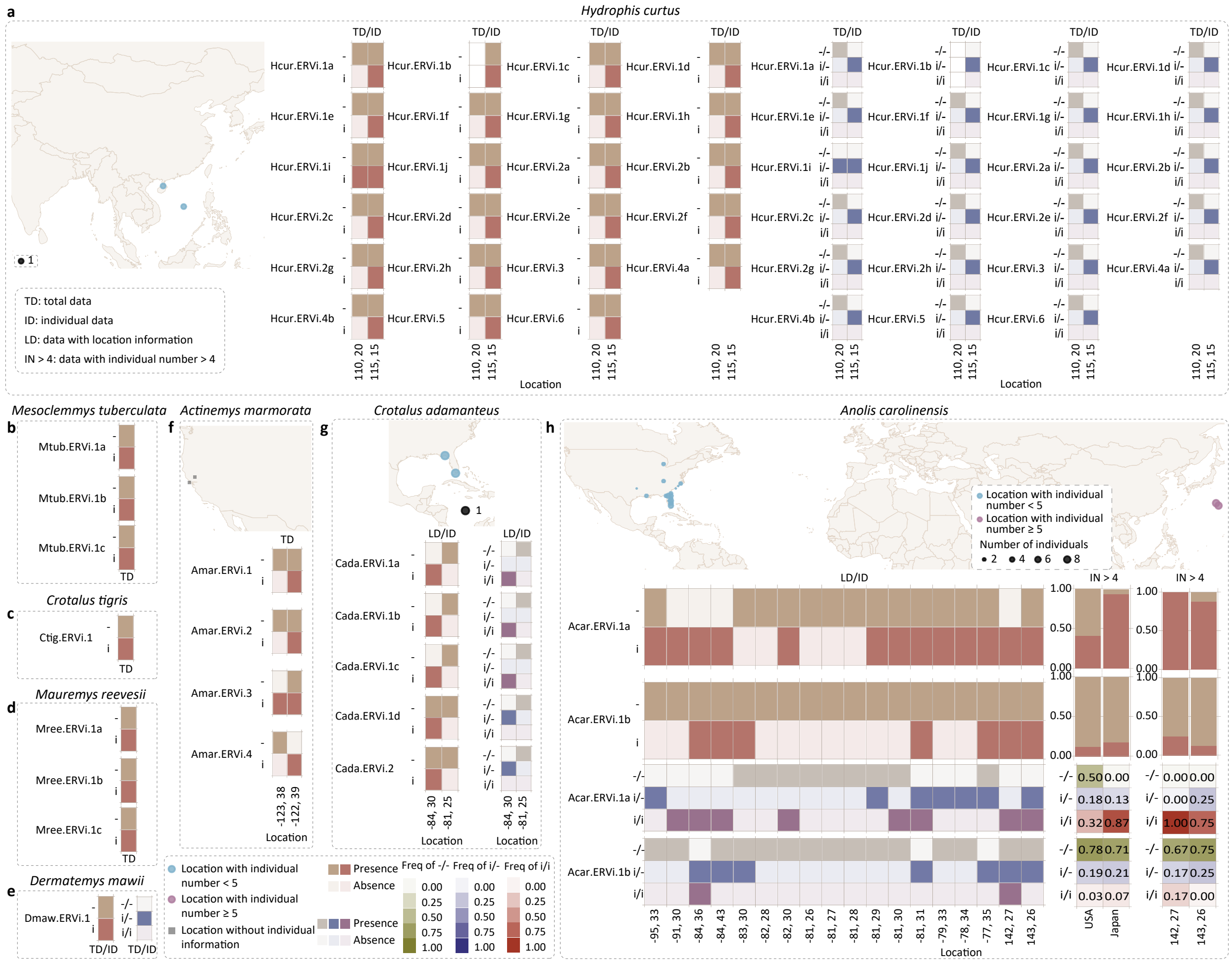

Supplementary Fig. 8 The geographic distribution and prevalence of ERVi in reptiles. a, *Hydrophis curtus*. **b**, *Mesoclemmys tuberculata*. **c**, *Crotalus tigris*. **d**, *Mauremys reevesii*. **e**, *Dermatemys mawii*. **f**, *Actinemys marmorata*. **g**, *Crotalus adamanteus*. **h**, *Anolis carolinensis*. For each species, diverse datasets were used to identify the presence (i) and absence (-) of ERVi insertion in a locus: TD (total data) include all the evidentiary genome sequencing data of individuals and samples without detailed individual information; ID (individual data) include evidentiary genome sequencing data for individuals; LD represents evidentiary genome sequencing data with location information. For each species with ID data, genotypes i/i, i/-, and -/- were shown. For each area grid cell (~ 12,321 km^2) or geographic region, when population genomics data for more than 4 individuals (IN > 4) are available, the frequency of genotype (i/i, i/- and -/-) were shown. Circle size indicates sample size in the corresponding location. Locations with individual number < and ≥ 5 are labeled in blue and purple, respectively. Locations without detail individual information are labeled with gray rectangles.

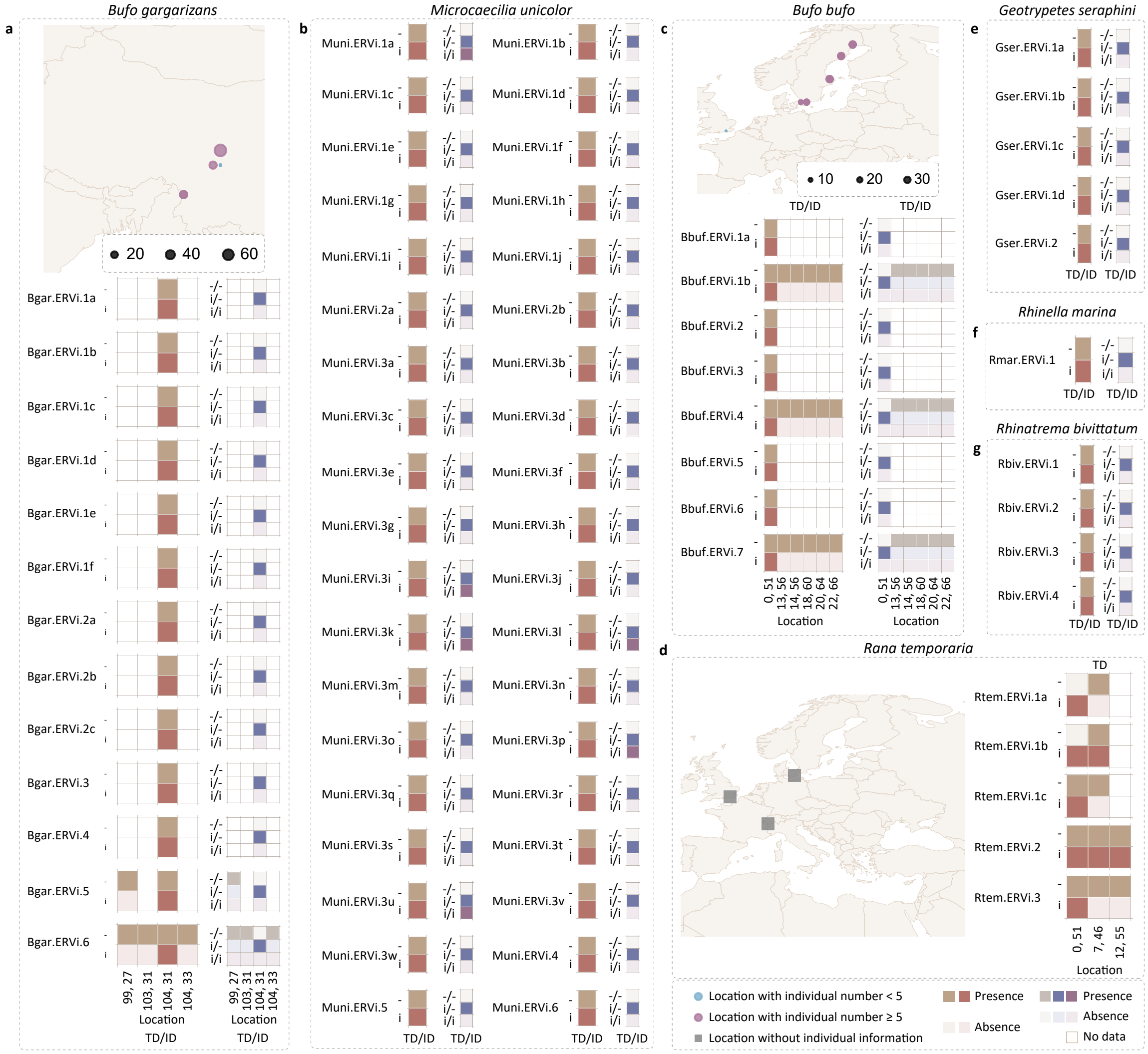

Supplementary Fig. 9 The geographic distribution and prevalence of ERVi in Amphibian. a, *Bufo gargarizans*. **b**, *Microcaecilia unicolor*. **c**, *Bufo bufo*. **d**, *Rana temporaria*. **e**, *Geotrypetes seraphini*. **f**, *Rhinella marina*. **g**, *Rhinatrema bivittatum*. For each species, diverse datasets were used to identify the presence (i) and absence (-) of ERVi insertion in a locus: TD (total data) include all the evidentiary genome sequencing data of individuals and samples without detailed individual information; ID (individual data) include evidentiary genome sequencing data for individuals or pooled individuals with specified sample size that can be inferred to be homozygous (i/i or -/-). For each species with ID data, genotypes i/i, i/-, and -/- were shown. Circle size indicates sample size in the corresponding location. Locations with individual number < and ≥ 5 are labeled in blue and purple, respectively. Locations without detail individual information are labeled with gray rectangles.

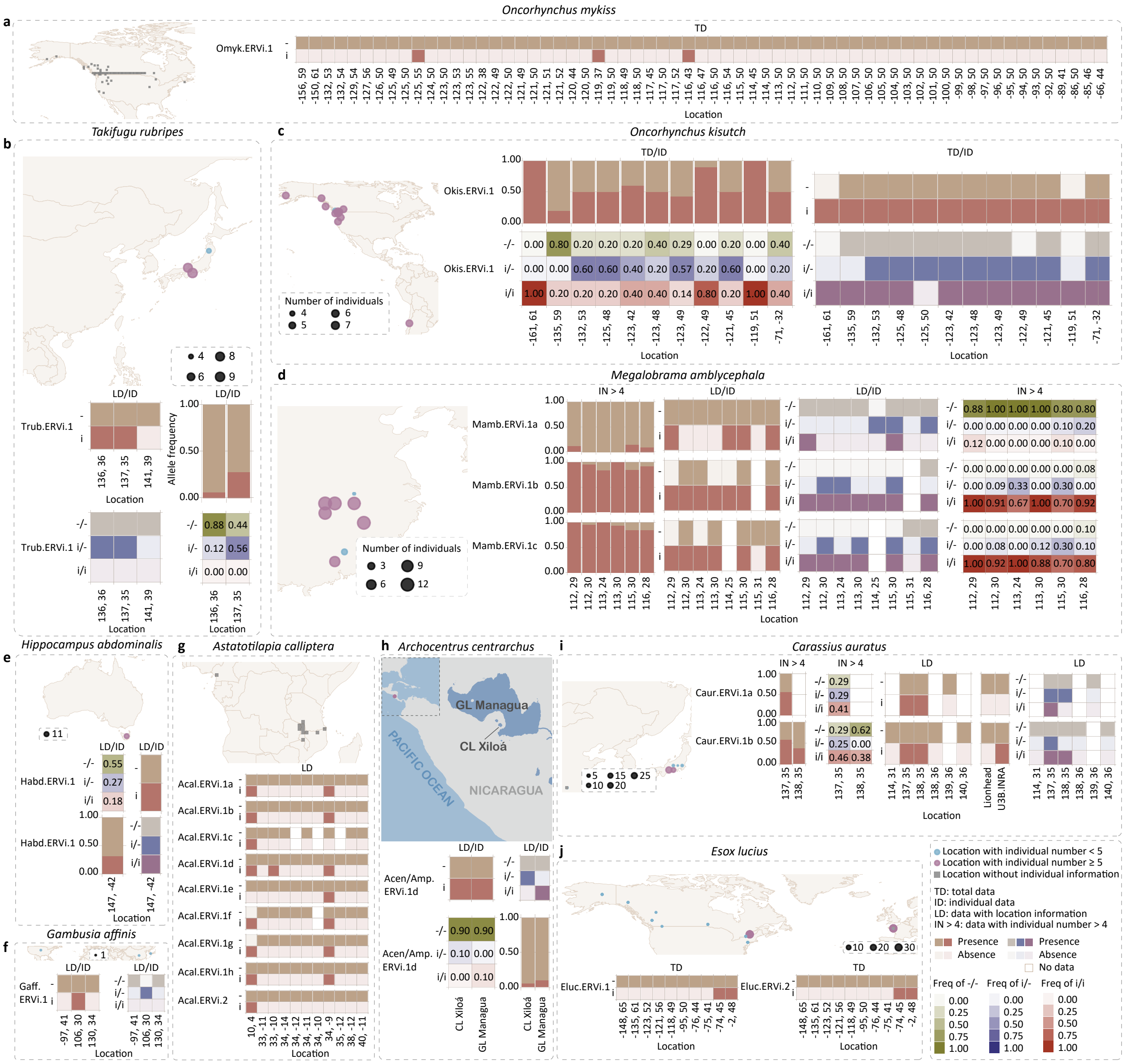

Supplementary Fig. 10 The geographic distribution and prevalence of ERVi in fishes. a, *Oncorhynchus mykiss*. **b**, T*akifugu rubripes*. **c**, *Oncorhynchus kisutch*. **d**, *Megalobrama amblycephala*. **e**, *Hippocampus abdominalis*. **f**, *Gambusia affinis*. **g**, *Astatotilapia calliptera*. **h**, *Archocentrus centrarchus*. **i**, *Carassius auratus*. **j**, *Esox lucius*. For each species, diverse datasets were used to identify the presence (i) and absence (-) of ERVi insertion in a locus: TD (total data) include all the evidentiary genome sequencing data of individuals and samples without detailed individual information; ID (individual data) include evidentiary genome sequencing data for individuals; LD represents evidentiary genome sequencing data with location information. For each species with ID data, genotypes i/i, i/-, and -/- were shown. For each species or area grid cell (~ 12,321 km^2), when population genomics data for more than 4 individuals (IN > 4) are available, the frequency of genotype (i/i, i/- and -/-) were shown. LD data used in (**i**) include individual

samples and multiple samples with location information but without individual information, from which homozygous genotypes (i/i or -/-) can be inferred. Circle size indicates sample size in the corresponding location. Locations with individual number $<$ and $\geq$ 5 are labeled in blue and purple, respectively. Locations without detail individual information are labeled with gray rectangles. Map in (**h**) was created using MAPCREATOR (https://api.mapcreator.io/). Abbreviations: crater lake (CL), and great lake (GL).

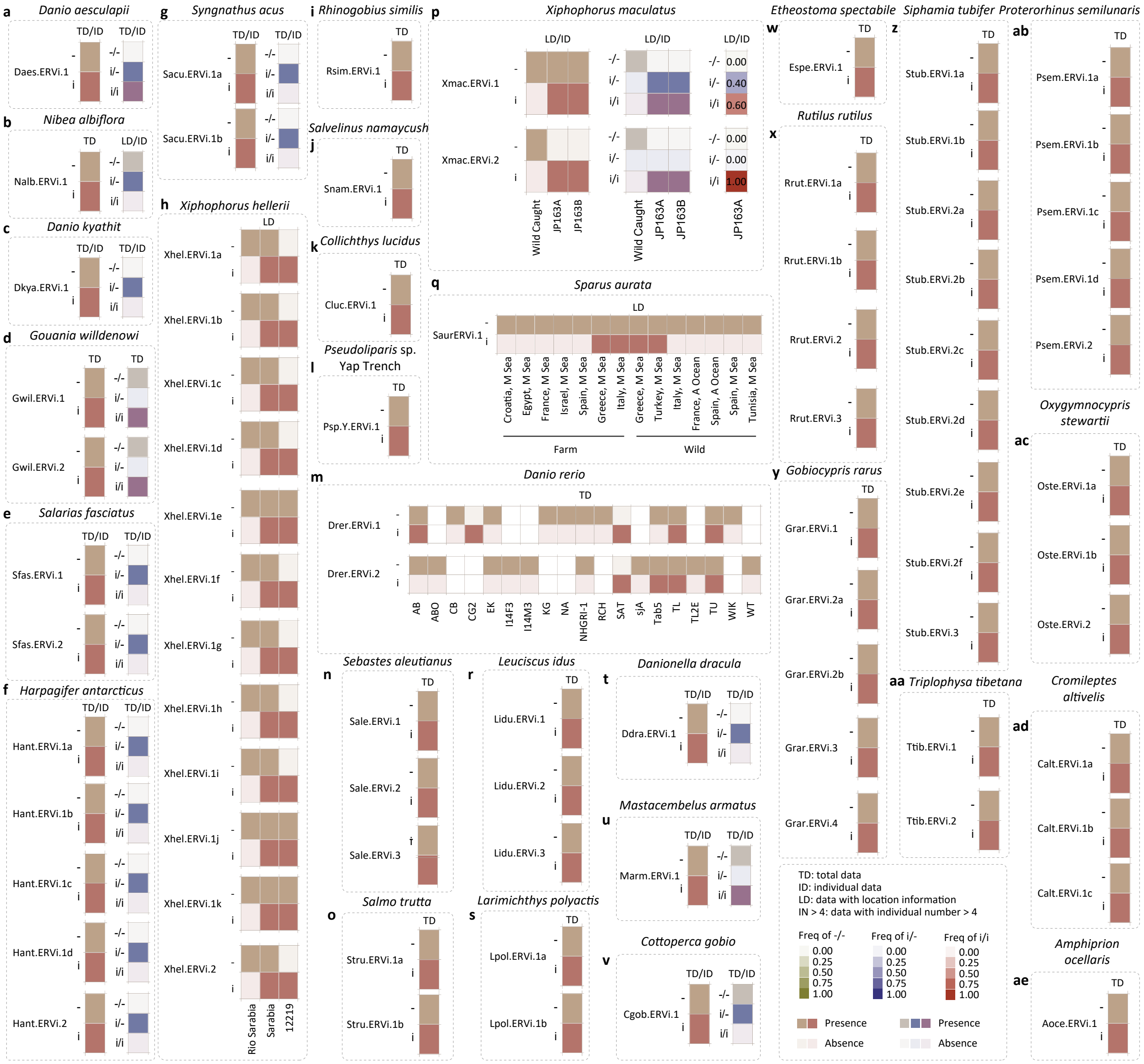

Supplementary Fig. 11 The geographic distribution and prevalence of ERVi in fishes. a, Danio aesculapii. **b**, Nibea albiflora. **c**, Danio kyathit. **d**, Gouania willdenowi. **e**, Salarias fasciatus. **f**, Harpagifer antarcticus. **g**, Syngnathus acus. **h**, Xiphophorus hellerii. **i**, Rhinogobius similis. **j**, Salvelinus namaycush. **k**, Collichthys lucidus. **l**, Pseudoliparis sp. Yap Trench. **m**, Danio rerio. **n**, Sebastes aleutianus. **o**, Salmo trutta. **p**, Xiphophorus maculatus. **q**, Sparus aurata. **r**, Leuciscus idus. **s**, Larimichthys polyactis. **t**, Danionella dracula. **u**, Mastacembelus armatus. **v**, Cottoperca gobio. **w**, Etheostoma spectabile. **x**, Rutilus rutilus. **y**, Gobiocypris rarus. **z**, Siphamia tubifer. **aa**, Triplophysa tibetana. **ab**, Proterorhinus semilunaris. **ac**, Oxygymnocypris stewartii. **ad**, Cromileptes altivelis. **ae**, Amphiprion ocellaris. For each species, diverse datasets were used to identify the presence (i) and absence (-) of ERVi insertion in a locus: TD (total data) include all the evidentiary genome sequencing data of individuals and samples without detailed individual information; ID (individual data) include evidentiary genome sequencing data for individuals; LD

represents evidentiary genome sequencing data with location information. For each species with ID data, genotypes i/i, i/-, and -/- were shown. For (**d**) pattern, TD data was used to show the presence and absence of i/i, i/-, and -/-, due to it is composed with individual samples and multiple samples without detailed individual information that can be inferred to be homozygous (i/i or -/-).

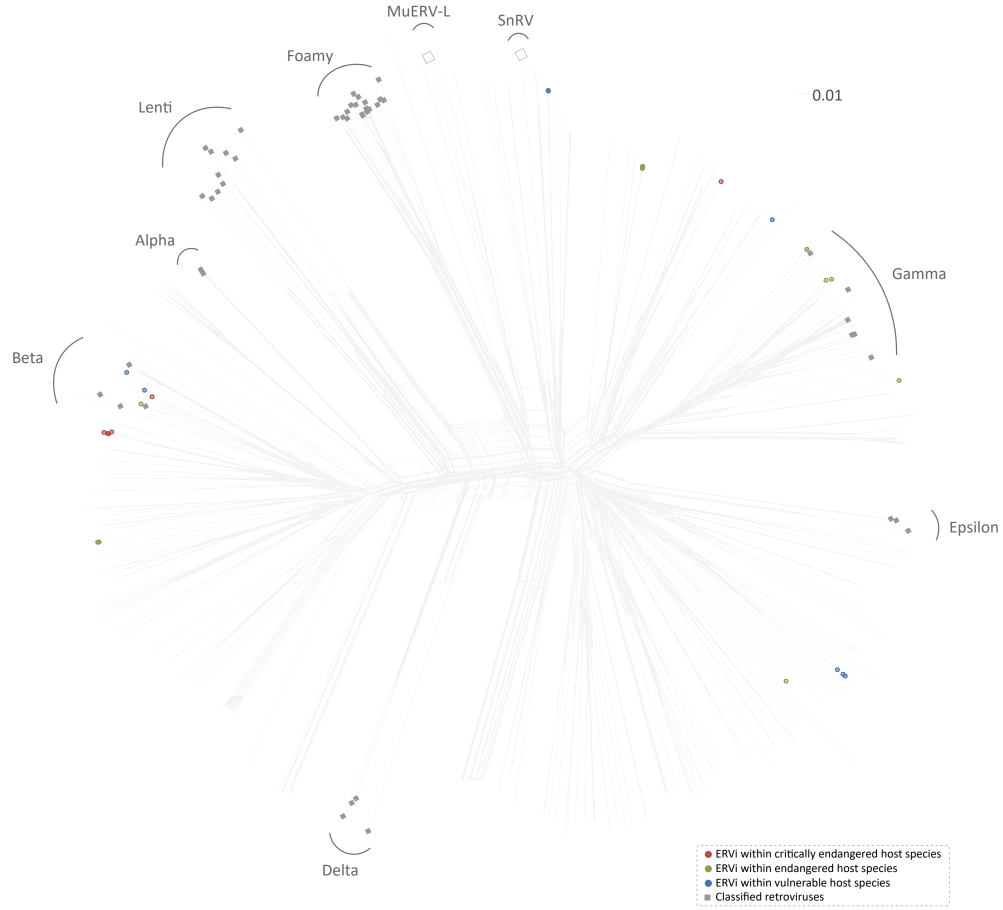

Supplementary Fig. 12 The phylogenetic network of ERVi vOTUs hosted by threatened species. Phylogenetic network of 412 ERVi elements, 48 retroviruses classified by ICTV, SnRV, and MuERV-L are reconstructed based on RT proteins. Filled circles and rectangles indicate ERVi and classified retroviruses, respectively. ERVi within threatened species are labeled in different colors. Abbreviations: viral operational taxonomic units (vOTUs), snakehead retrovirus (SnRV), and murine retrovirus endogenous retrovirus-L (MuERV-L).
