# Supplementary material for: Genome mining shows that retroviruses are pervasively invading vertebrate genomes
Source: Nat Commun. 2023 Aug 17;14:4968. doi: 10.1038/s41467-023-40732-w (PMC10435555; doi:10.1038/s41467-023-40732-w)
Supplement: Supplementary file 3 — Reporting Summary [file 41467_2023_40732_MOESM3_ESM.pdf]

nature portfolio

Corresponding author(s): Guan-Zhu Han

Last updated by author(s): Jul 14, 2023

Reporting Summary

Nature Portfolio wishes to improve the reproducibility of the work that we publish. This form provides structure for consistency and transparency in reporting. For further information on Nature Portfolio policies, see our Editorial Policies and the Editorial Policy Checklist.

Statistics

For all statistical analyses, confirm that the following items are present in the figure legend, table legend, main text, or Methods section.

n/a	Confirmed	
☐	☒	The exact sample size (*n*) for each experimental group/condition, given as a discrete number and unit of measurement
☒	☐	A statement on whether measurements were taken from distinct samples or whether the same sample was measured repeatedly
☒	☐	The statistical test(s) used AND whether they are one- or two-sided<br>*Only common tests should be described solely by name; describe more complex techniques in the Methods section.*
☒	☐	A description of all covariates tested
☒	☐	A description of any assumptions or corrections, such as tests of normality and adjustment for multiple comparisons
☐	☒	A full description of the statistical parameters including central tendency (e.g. means) or other basic estimates (e.g. regression coefficient) AND variation (e.g. standard deviation) or associated estimates of uncertainty (e.g. confidence intervals)
☒	☐	For null hypothesis testing, the test statistic (e.g. *F*, *t*, *r*) with confidence intervals, effect sizes, degrees of freedom and *P* value noted<br>*Give P values as exact values whenever suitable.*
☒	☐	For Bayesian analysis, information on the choice of priors and Markov chain Monte Carlo settings
☐	☒	For hierarchical and complex designs, identification of the appropriate level for tests and full reporting of outcomes
☒	☐	Estimates of effect sizes (e.g. Cohen's *d*, Pearson's *r*), indicating how they were calculated

Our web collection on statistics for biologists contains articles on many of the points above.

Software and code

Policy information about availability of computer code

Data collection	No software was used to collect data in this study.
Data analysis	All softwares (LTRharvest implemented in GenomeTools v1.5.10, MAFFT v7.402, MAFFT v7.475, FastTree v2.1.10, ORFfinder v0.4.3, Gephi v0.9.7, mcl 14-137, trimAl v1.2, SplitsTree v4.18.3, R v4.2.1) and algorithms (tBLASTn algorithm and BLASTn algorithm implemented in BLAST 2.12.0+, MCL algorithm implemented in mcl 14-137, community detection algorithm implemented in Gephi v0.9.7, Conserved domain (CD) search against database CDD v3.20-59693 PAAMs, HMMER 3.2.1, dplyr package v1.1.2, boot package v1.3.28.1, caper package v1.0.2, RVAideMemoire package v0.9.83) used to analyze data in this study are open source.

For manuscripts utilizing custom algorithms or software that are central to the research but not yet described in published literature, software must be made available to editors and reviewers. We strongly encourage code deposition in a community repository (e.g. GitHub). See the Nature Portfolio guidelines for submitting code & software for further information.

Data

Policy information about availability of data

All manuscripts must include a data availability statement. This statement should provide the following information, where applicable:

- Accession codes, unique identifiers, or web links for publicly available datasets
- A description of any restrictions on data availability
- For clinical datasets or third party data, please ensure that the statement adheres to our policy

No new data were generated in support of this research. All data used or generated in this study were provided in Supplementary Data or repository. Vertebrate

genomes and SRA data used in this study were retrieved from NCBI, and the accession numbers were provided in Supplementary Data 1 and 4, respectively. The sequences of all the ERVi identified in this study have been deposited in Figshare (doi: 10.6084/m9.figshare.23653254). Sequence alignment of RT protein sequences used for phylogenetic analysis is provided as Supplementary Data 11.

Research involving human participants, their data, or biological material

Policy information about studies with human participants or human data. See also policy information about sex, gender (identity/presentation), and sexual orientation and race, ethnicity and racism.

Reporting on sex and gender	There are no relevant problems in this study.
Reporting on race, ethnicity, or other socially relevant groupings	There are no relevant problems in this study.
Population characteristics	There are no relevant problems in this study.
Recruitment	There are no relevant problems in this study.
Ethics oversight	There are no relevant problems in this study.

Note that full information on the approval of the study protocol must also be provided in the manuscript.

Field-specific reporting

Please select the one below that is the best fit for your research. If you are not sure, read the appropriate sections before making your selection.

☐ Life sciences ☐ Behavioural & social sciences ☒ Ecological, evolutionary & environmental sciences

For a reference copy of the document with all sections, see nature.com/documents/nr-reporting-summary-flat.pdf

Ecological, evolutionary & environmental sciences study design

All studies must disclose on these points even when the disclosure is negative.

Study description	In this study, genomics data of 2,004 vertebrate species were used to systemically mine retroviruses that are invading their host genomes (ERVi). Besides, the pattern and nature of ERVi in the historical and biogeographical context of their hosts, such as the generation of model organisms, sympatric speciation, and domestication were characterized. The results of this study expands the diversity of retrovirus and elongates the list of retroviruses potentially circulating in the wild. The discovery of hundreds of ERVi at human-animal interface provides valuable resource for virus surveillance and zoonotic risk assessment.
Research sample	To confirm that these ERVi candidates are invading their host genomes, we performed insertional polymorphism analyses using almost all the available genome-scale sequence read archive (SRA) data (4,316) from 217 host species in NCBI (~168.12 tera-bases in total). Considering the difference of sequencing coverage, one meaningful read was used as cut-off to identify the presence or absence of ERVi candidates in the host genomes. SRA data that provide evidence for insertional polymorphism of ERVi were considered as evidentiary sequencing data.
Sampling strategy	The number of datasets for each species depends on the number of the publicly available genome-scale sequence read archive (SRA) data in NCBI.
Data collection	Jianhua Wang collected the data and Excel was used to gather all these data.
Timing and spatial scale	The genome sequencing data used in this study were collected from NCBI during Mar 19th 2021 to Aug 25th 2022. Data collection almost run through the entire research.
Data exclusions	Low-quality genome sequencing data were excluded in this study.
Reproducibility	The results of this study are repetable.
Randomization	All the representative vertebrate genomes were retrieved from NCBI. To confirm that these ERVi candidates are invading their host genomes, we performed insertional polymorphism analyses using almost all the available genome-scale sequence read archive (SRA) data (4,316) from 217 host species in NCBI (~168.12 tera-bases in total). Considering the difference of sequencing coverage, one meaningful read was used as cut-off to identify the presence or absence of ERVi candidates in the host genomes. SRA data that provide evidence for insertional polymorphism of ERVi were considered as evidentiary sequencing data. For species (e.g. M. musculus) with too many public SRA data available in NCBI, we randomly selected some SRA data to perform the insertional polymorphism analyses for them.
Blinding	This is a large-scale research which aims to understanding the distribution and prevalence of invading endogenous retroviruses, so we prefer to collect and use genome sequencing data as many as possible. Thus, we think there is no blinding issue in this study.

Did the study involve field work? ☐ Yes ☒ No

Reporting for specific materials, systems and methods

We require information from authors about some types of materials, experimental systems and methods used in many studies. Here, indicate whether each material, system or method listed is relevant to your study. If you are not sure if a list item applies to your research, read the appropriate section before selecting a response.

Materials & experimental systems

n/a	Involved in the study
☒	☐ Antibodies
☒	☐ Eukaryotic cell lines
☒	☐ Palaeontology and archaeology
☒	☐ Animals and other organisms
☒	☐ Clinical data
☒	☐ Dual use research of concern
☒	☐ Plants

Methods

n/a	Involved in the study
☒	☐ ChIP-seq
☒	☐ Flow cytometry
☒	☐ MRI-based neuroimaging
