# Supplementary material for: Genome mining shows that retroviruses are pervasively invading vertebrate genomes
Source: Nat Commun. 2023 Aug 17;14:4968. doi: 10.1038/s41467-023-40732-w (PMC10435555; doi:10.1038/s41467-023-40732-w)
Supplement: Supplementary file 4 — Description of Additional Supplementary Files [file 41467_2023_40732_MOESM4_ESM.pdf]

## Description of Additional Supplementary Files

File Name: Supplementary Data 1

Description: The detailed information for 2,004 vertebrate species used in this study

File Name: Supplementary Data 2

Description: Queries used in this study

File Name: Supplementary Data 3

Description: The length cut-off used for detecting intact ORFs

File Name: Supplementary Data 4

Description: Genome sequencing data used in insertional polymorphism analyses

File Name: Supplementary Data 5

Description: The detailed information for 412 ERVi

File Name: Supplementary Data 6

Description: Data used in hierarchical clustering

File Name: Supplementary Data 7

Description: The results of hierarchical clustering

File Name: Supplementary Data 8

Description: Data used in Fig. 2d

File Name: Supplementary Data 9

Description: Data used in Fig. 2e

File Name: Supplementary Data 10

Description: The documented association routes for contact between vertebrates and humans
